# Relationship between brain-derived neurotrophic factor and immune function during dietary supplement treatment of elderly with Alzheimer’s dementia

**Published:** 2020-01-29

**Authors:** Jordan Stillman, Alicia Martin, Maria-Jose Miguez, H. Reginald McDaniel, Janet Konefal, Judi M. Woolger, John E. Lewis

**Affiliations:** ^1^Department of Psychiatry and Behavioral Sciences, Miami, Florida; ^2^Department of School of Integrated Science and Humanity, Florida International University, Miami, Florida; ^3^Department of Fisher Institute for Medical Research, Grand Prairie, Texas, United States; ^4^Department of Family Medicine and Community Health, Miami, Florida; ^5^Department of Medicine, University of Miami Miller School of Medicine, Miami, Florida

**Keywords:** aloe polymannose, Alzheimer’s dementia, brain-derived neurotrophic factor, CD95+CD3, dietary supplementation, epidermal growth factor, immune function, polysaccharide, vascular endothelial growth factor

## Abstract

**Background and Aim:**

The objective of the present study was to investigate the relationships among pro-brain-derived neurotrophic factor (BDNF) and mature BDNF and immune functioning during aloe polymannose multinutrient complex (APMC) treatment in persons with moderate to severe Alzheimer’s dementia (AD).

**Materials and Methods:**

An open-label trial of 12 months was used to execute the study. Thirty-four adults with AD were enrolled and consumed four teaspoons/day of APMC for 12 months. Subjects were assessed at baseline and 12 months follow-up for proBDNF and BDNF and cytokines, growth factors, T-cell and B-cell subsets, and complete blood count to measure immune functioning. All biomarkers were intercorrelated.

**Results:**

Several relationships were identified between proBDNF, BDNF, and BDNF/proBDNF ratio and immune function at 12 months, particularly BDNF with vascular endothelial growth factor (VEGF) (*r*=0.55, *P*=0.03), epidermal growth factor (EGF) (*r*=0.74, *P*=0.001), and CD95+CD3+ (%) (*r*=−0.64, *P*=0.03) and proBDNF with VEGF (*r*=0.64, *P*=0.02), EGF (*r*=0.86, *P*<0.001), and CD16+56+ (%) (*r*=−0.78, *P*<0.01). Other correlations were noted for various immune function variables with BDNF, proBDNF, and/or BDNF/proBDNF ratio at baseline and 12 months. Dichotomizing subjects on BDNF above and below 5000 pg/mL revealed additional relationships with platelets and neutrophils.

**Conclusions:**

The associations between BDNF and proBDNF and various immune markers, such as VEGF, EGF, and CD95+CD3+ ratio, provide insight into the link between neurological function and the immune system. These relationships were even stronger in response to APMC treatment, which lends support to previous findings showing improved immune function after dietary supplementation.

**Relevance for Patients:**

AD patients have conventional treatment options with limited efficacy for counteracting the deleterious effects of the disease on neurological function. The link between neurological and immune function has been understudied in this population. Overall, our results showed a significant beneficial relationship between immune and neurological function, particularly in response to 12 months of treatment with an all-natural polysaccharide-based dietary supplement that is a known immunomodulator. Thus, the use of this dietary supplement may benefit these patients by simultaneously improving immune and neurological function.

## 1. Introduction

Brain-derived neurotrophic factor (BDNF) is one of the most recognized neuromodulators in the human brain. It plays a critical role in neurosynaptic function, apoptosis, plasticity, long-term potentiation, learning memory processes, and higher-order thinking [1-6]. More specifically, it is active in the synapses of the central nervous system (CNS), where it works to promote growth, maturation, neuronal survival, and synaptic plasticity [7]. This makes it an important regulator of long-term potentiation and thus learning and memory. The biochemical level to which BDNF is involved in this process is still being elucidated. However, studies have demonstrated its role in initial memory consolidation through vital cell growth pathways, such as the regulation of nuclear factor kappa B [8-11]. For decades, the assumption was that proBDNF did not have a physiological function, but very recently, Je *et al*. demonstrated that proBDNF interacts with the low-affinity receptor p75, while mature BDNF signals through its high-affinity tropomyosin-related kinase B receptor [12]. As a result, they frequently exert opposite neuronal functions [13]. Therefore, it is critical to measure both and the ratio between the two, when evaluating their potential roles in studies of dementia.

Thus, it is plausible to posit that BDNF could play a role in either the pathological mechanism or treatment of diseases that affect cognition and memory through cell death, such as Alzheimer’s dementia (AD). AD is the most common cause of dementia and one of the leading causes of morbidity and mortality in the aging population [14]. The relationship between BDNF and certain pro-inflammatory cytokines, such as tumor necrosis factor (TNF)-α, transforming growth factor-β1, interleukin (IL)-6, IL-8, and IL-1β, has been well-documented [15,16]. In AD, increasing levels of β-amyloid plaques and τ proteins lead to synaptic loss, neuronal damage, and finally neuronal death [17-19], which is otherwise known as neuroinflammation mediated by the microglia. Neuroinflammation causes the microglia to overproduce the same pro-inflammatory cytokines, for example, TNF-α, interferon (IFN), IL-1β, and IL-6, that are linked to the underproduction of BDNF in AD patients [18,20]. This increasing neuroinflammation along with reduced BDNF is associated with the progression and proliferation of symptoms in late stages of AD [18].

We have previously shown that dietary supplementation can reduce inflammation in several populations, including those with AD [21-24]. Most relevant to the current analysis, we conducted a 12-month intervention in persons with moderate-to-severe AD, showing that an aloe polymannose multinutrient complex (APMC) improved their immune function (according to TNF-α, IL-2, IL-4, and vascular endothelial growth factor [VEGF]) and increased the production of adult stems according to CD14+ by just under 300% [21]. Simultaneously, we have shown that cognitive function is related to BDNF, particularly at a level 5000+ pg/mL [25]. Many of the nutrients in APMC, including polysaccharides, antioxidants, and omega-3 fatty acids, have individual benefits on BDNF and/or markers of inflammation [17-20,26].

Therefore, we theorize that the benefits of APMC on the relationship between BDNF and cognitive functioning could extend to inflammation and immune function. To support this hypothesis in the current study, our primary objectives are to (1) assess the relationships among proBDNF and BDNF with a battery of cytokines, growth factors, and T cell and B cell subsets before and after 12 months of supplementation with APMC and (2) evaluate if the 5000 pg/mL cutoff value for BDNF that we previously found relevant to discriminate levels of cognitive functioning in this AD population will be applicable/useful for distinguishing different values in the biomarkers of interest. These objectives will allow us to evaluate if BDNF is related to inflammation and immune function before and after supplementation with APMC in a sample of subjects with moderate-to-severe AD.

## 2. Materials and Methods

### 2.1. Subjects

Participants (*n*=34) were recruited from the Miami Jewish Health Systems facility from 2008 to 2011. The study was conducted with the approval of the Stein Gerontological Institute Institutional Review Board for human subjects research, and each subject and the primary caregiver signed informed consent before participating in the study. This study was not listed with clinicaltrials.gov, as that was not a policy requirement at that time. Inclusion criteria were: (a) ≥60 years of age, (b) a clinical diagnosis by the study psychiatrist of probable moderate-to-severe AD for at least 1 year, (c) a score of between 10 and 26 on the Mini-Mental State Examination [27], (d) sufficient vision and hearing to comply with study procedures, and (e) able to provide informed consent by the subject or primary caregiver. Exclusion criteria were: (a) Diagnoses of delirium or depression, (b) currently participating in another nutritional supplement study, and (c) a known allergy to eating shrimp. Each participant was evaluated by the study psychiatrist before enrollment in the study to verify the diagnosis of AD according to the standards and criteria of the diagnostic and statistical manual of mental disorders. Subjects were not required to change their medication regimen to participate in the study and continued to take their daily medication as prescribed by their treating physician.

### 2.2. Intervention

The polysaccharide-based multinutrient formula used in this study is a nutritional supplement that has been sold by several commercial entities for over 20 years. According to the company’s literature (www.wellnessquest.org), APMC contained stabilized rice bran, larch tree fiber and larch tree soluble extract, cysteine, soy lecithin, aloe leaf powder (BiAloe^®^), inositol hexaphosphate, dioscorea powder, omega-3 spherules, citric acid, glucosamine, cherry tart powder, and ultraterra calcium aluminosilicate. The final product was a powder, packaged in 300 g containers, which dissolves readily in any liquid. All participants consumed one teaspoon orally of the APMC 4 times per day (with three meals and before bedtime). The primary caregiver was shown how to administer the APMC at the baseline assessment, and the first dose was given to the participant at our facility to ensure compliance with the method and to monitor for any complications or adverse effects.

### 2.3. Sample collection and processing

Venous blood was obtained at baseline and 12 months from all participants to assess changes in the biomarkers. To obtain platelet-poor plasma (PPP), blood samples were collected in EDTA-coated tubes (plasma) (BD Diagnostics, Franklin Lakes, NJ), stored on ice, and delivered to the laboratory within 2 h of data collection. Plasma was separated by centrifugation at 40°C for 15 min at 1500× *g*. The plasma was again recentrifuged at 10,000× *g* and aliquots of PPP were stored in polypropylene tubes at −80°C until assayed. All specimens were subjected to complete blood cell counts and auto five-part differential count determinations by a fully automated Coulter AcT5 hematology analyzer (Beckman Coulter, Fullerton, CA). Flow cytometric enumeration of T, B, and natural killer (NK) cell subsets was performed on a 4-color flow cytometer, FACSCalibur (BD Biosciences, San Jose, CA), and the different cell populations were analyzed using Cellquest Pro Software (version 5.2, BD Biosciences, San Jose, CA).

Peripheral blood mononuclear cells (PBMC) were isolated by Ficoll-Hypaque gradient centrifugation. PBMC were recovered from the gradient interface and washed in phosphate-buffered saline. Blood was diluted with 1:1 RPMI 1640 (Gibco, Grand Island, NY), layered over Ficoll-Hypaque solution (Pharmacia, Piscataway, NJ), and centrifuged for 30 min at 1500 rpm at ambient temperature. The PBMC were collected, washed with RPMI 1640, and counted and assessed for viability in Trypan blue dye. Plasma for cytokine detection was separated and stored at −80°C until used.

### 2.4. BDNF and proBDNF sample processing

Circulating levels of BDNF were selected because prior studies have demonstrated that although different from those in the cerebrospinal fluid (CSF), they are correlated with CSF measures in other CNS diseases [28]. PPP BDNF and proBDNF levels were measured using a commercially available ELISA kit (R&D System) according to the manufacturer’s instructions and were calculated based on a standard curve. The minimum detectable concentration of BDNF is typically <62 pg/mL. The repeatability of the BDNF ELISA, as measured by intra-assay precision, was 6% and the reproducibility as measured by inter-assay precision was 9%. Coefficient of variation was 7.9 (CV% = SD/mean×100%).

### 2.5. Multiplex cytokine and growth factor testing

Cytokine and growth factor levels in plasma specimens were measured using a biochip array system, Evidence Investigator^™^ (Randox Laboratories Ltd., Crumlin, UK) as reported previously [29]. The testing platform consisted of biochips secured in the base of a well placed in a carrier holding nine biochips in a 3×3 format. Each biochip was coated with the capture antibodies specific for each of the 12 cytokines and growth factors (IL-2, IL-4, IL-6, IL-8, IL-10, IL-1α, IL-1β, IFN-γ, TNF-α, monocyte chemotactic protein [MCP]-1, VEGF, and epidermal growth factor [EGF]) on a particular test region. A sandwich chemiluminescent assay was performed with 100 µL plasma using reagents (including the calibrators and controls) and protocols supplied by the same manufacturer. The light signal generated from each of the test regions on the biochip was detected using a charge-coupled detector camera and imaging system and compared with a calibration curve generated with known standards during the same run. All specimens were run in duplicate, and the concentration of each cytokine present in each plasma specimen was calculated from the standard curve and reported in pg/mL.

### 2.6. Statistical analysis

Data were analyzed using IBM SPSS 24 (IBM, Chicago, IL) for Windows. Frequency and descriptive statistics were calculated on all variables. The relationship between BDNF, proBDNF, and BDNF/proBDNF ratio and cytokines, growth factors, and T-cell and B-cell subsets were examined at baseline and 12 months follow-up with Pearson product-moment correlations. Differences in baseline and 12-month values on BDNF, proBDNF, and the biomarkers were calculated to assess for correlation among the difference scores. The sample was split by BDNF at 5000 pg/mL based on our prior findings to assess differences in inflammation and immune functioning between the two groups. The criterion for statistical significance was α=0.05.

## 3. Results

### 3.1. Sociodemographics and descriptives for all biomarkers

The sample comprised 82% females (*n*=28) and 18% males (*n*=6) with a mean age of 79.9±8.4 years. Table 1 displays all sociodemographic variables for the study sample, and Tables 2-4 show all biomarkers at baseline and 12 months, including proBDNF, BDNF, cytokines, growth factors, and T-cell and B-cell subsets, which were previously published [21,25]. At baseline, BDNF and proBDNF were not correlated with age, sex, race/ethnicity, marital status, educational attainment, or years diagnosed with AD.

**Table 1:** Sociodemographic characteristics of the sample.

Variable	Category	Baseline assessment (*n*=34) (%)
Age	-	M=79.9 (SD=8.4; R=60, 98)

Gender	Male	6 (17.6)

	Female	28 (82.4)

Race/ethnicity	White, non-Hispanic	10 (29.4)

	Black, non-Hispanic	3 (8.8)

	Hispanic	21 (61.7)

Education	Up to high school	23 (67.6)

	Some post-high school training	3 (8.8)

	College graduate	4 (11.8)

	Master’s degree or higher	4 (11.8)

Marital status	Never married	2 (5.9)

	Married	15 (44.1)

	Widowed	13 (38.2)

	Divorced	4 (11.7)

Years diagnosed with Alzheimer’s disease	-	M=3.2 (SD=2.0; R=1, 11)

M: Mean, SD: Standard deviation, R: Range

**Table 2:** BDNF, proBDNF, cytokines, and growth factors at baseline and 12 months follow-up.

Variable	Baseline	12 months
BDNF (pg/mL)	5673.7±4003.5 (939, 14,802)	6312.8±4198.9 (1453, 15,306)

proBDNF (pg/mL)	6108.8±1480.7 (3497, 9552)	5799.4±993.2 (4574, 8267)

BDNF/proBDNF ratio	1.01±0.71 (0.25, 2.61)	1.15±0.56 (0.28, 1.85)

IL-2 (pg/mL)*	6.4±4.6 (0, 19.1)	4.3±6.7 (0, 29.8)

IL-4 (pg/mL)*	0.94±1.42 (0, 4.3)	0.25±0.89 (0, 3.8)

IL-6 (pg/mL)	5.2±7.6 (0, 37.7)	5.1±11.6 (0, 56.5)

IL-8 (pg/mL)	7.4±11.3 (0, 62.9)	11.5±34.3 (0, 174.0)

IL-10 (pg/mL)	0.34±0.62 (0, 2.4)	0.97±3.65 (0, 18.4)

IL-1α (pg/mL)	0.21±0.31 (0, 0.75)	0.13±0.60 (0, 3.0)

IL-1β (pg/mL)	1.8±2.3 (0, 8.6)	4.0±10.8 (0, 44.0)

IFN-γ (pg/mL)	0.91±1.79 (0, 7.4)	0.47±1.21 (0, 5.0)

TNF-α (pg/mL)*	2.8±1.6 (0, 5.0)	1.7±1.4 (0, 4.0)

MCP-1 (pg/mL)	127.3±62.8 (45.6, 302.5)	122.1±44.7 (47.6, 212.6)

VEGF (pg/mL)*	50.4±31.6 (12.8, 150.1)	31.2±22.6 (0, 79.9)

EGF (pg/mL)	10.1±14.0 (0, 53.7)	10.1±15.0 (0, 68.6)

* Values are significantly different (*P*<0.05) from baseline to 12 months; mean±standard deviation (minimum, maximum). EGF: Epidermal growth factor, VEGF: Vascular endothelial growth factor, MCP: Monocyte chemotactic protein, TNF: Tumor necrosis factor, IFN: Interferon, IL: Interleukin, BDNF: Brain-derived neurotrophic factor

**Table 3:** T-cell subsets at baseline and 12 months follow-up.

Variable	Baseline	12 months
WBC (cells/uL)	6891.2±2168.1 (2700, 11,600)	6652.0±2089.9 (3300, 10,800)

Lymphs (%)	28.3±7.6 (14.3, 41.4)	29.9±8.0 (18.7, 48.1)

CD45+ (cells/uL)	1897.2±709.3 (840, 3828)	1936.2±625.6 (780, 3402)

CD3+ (%)	71.3±9.5 (46, 86)	71.8±8.6 (54, 88)

CD3+ (Cells/uL)	1342±503.7 (551, 2666)	1381.4±470.3 (507, 2888)

CD3+CD4+ (%)	46.6±9.8 (26.3, 68)	46.6±9.7 (31, 71)

CD3+CD4+ (cells/uL)	861.7±293.3 (337, 1514)	881.1±291.9 (398, 1592)

CD3+CD8+ (%)	24.4±9.8 (7, 43)	24.7±9.9 (2, 44)

CD3+CD8+ (cells/uL)	476.2±292.6 (97, 1418)	489.4±279 (32, 1238)

B cells CD19+ (%)	9.4±5.7 (0.9, 30)	9.2±6.4 (1, 28)

B cells CD19+ (cells/uL)	193.4±191.4 (14, 1093)	197.7±170.5 (16, 772)

NK cells CD16+56 (%)	18.5±9.1 (5.5, 46)	17.8±7.3 (4, 31)

NK cells CD16+56 (cells/uL)	346.2±203.8 (92, 838)	338.4±167.2 (71, 676)

CD3+CD4+/CD3+CD8+ ratio*	2.5±2.0 (0.6, 9.7)	3.4±6.8 (0.7, 35.5)

* Values are significantly different (*P*<0.05) from baseline to 12 months; values are mean±standard deviation (minimum, maximum). WBC: White blood cells, NK: Natural killer

**Table 4:** CD14, CD34, CD90, and CD95 subsets at baseline and 12-month follow-up.

Variable	Baseline	12 months
CD34+ (%)	24.5±20.5 (0.3, 66.3)	13.1±18.2 (0.5, 56.9)

CD34+ (cells/uL)	441.6±378.8 (5, 1251)	231.1±322.3 (6, 990)

CD90+ (%)*	9.3±15.1 (0.9, 48.1)	1.2±1.7 (0.1, 6.1)

CD90+ (cells/uL)*	154.4±251.0 (17, 775)	23.5±38.0 (2, 133)

CD95+CD3+ (%)*	52.5±19.9 (7.1, 85.8)	15.6±16.6 (1.1, 65.5)

CD95+CD3+ (cells/uL)*	937.7±396.9 (134, 1586)	305.7±361.8 (15.0, 1427)

CD95+CD34+ (%)*	24.9±12.6 (5.7, 47.7)	4.3±10.3 (0.1, 39.5)

CD95+CD34+ (cells/uL)*	427.3±198.8 (127, 796)	87.3±222.7 (2, 861)

CD95+CD90+ (%)*	7.5±12.0 (0.8, 40.9)	1.7±2.7 (0, 9.4)

CD95+CD90+ (cells/uL)	125.3±196.0 (13, 659)	31.1±44.2 (0, 129)

CD14+ (%)*	10.3±5.0 (5.5, 17.6)	39.8±22.6 (5.2, 80.0)

CD14+CD34+ (%)	7.5±14.3 (0, 38.4)	3.4±4.9 (0.3, 18.5)

CD14+CD90+ (%)*	18.0±16.4 (1.9, 61.3)	2.4±3.6 (0.1, 14.3)

CD14+CD95+ (%)*	77.5±19.0 (23.7, 97.3)	26.2±19.2 (4.9, 82.7)

* Values are significantly different (*P*<0.05) from baseline to 12 months; values are mean±standard deviation (minimum, maximum).

### 3.2. The relationship between BDNF and immune function

At baseline, BDNF was correlated with IL-10 (*r*=0.45, *P*=0.03), VEGF (*r*=0.40, *P*=0.05), and EGF (*r*=0.65, *P*=0.001). BDNF was also correlated with CD3+ (%) lymphocytes (*r*=−0.42, *P*=0.04) and CD14+CD34+ (%) monocytes (*r*=0.89, *P*=0.04), and trends were noted for CD16+56+ (cells/µL) (*r*=0.38, *P*=0.07) and CD16+56+ (%) (*r*=0.38, *P*=0.07). At 12 months follow-up, BDNF was correlated with VEGF (*r*=0.55, *P*=0.03) and EGF (*r*=0.74, *P*=0.001), and a trend was noted for MCP-1 (*r*=0.44, *P*=0.09). BDNF was correlated with CD95+CD3+ (%) lymphocytes (*r*=−0.64, *P*=0.03), CD95+CD3+ (cells/µL) lymphocytes (*r*=−0.59, *P*=0.04), and CD14+CD95+ (%) monocytes (*r*=−0.68, *P*=0.02). BDNF was correlated with mean corpuscular hemoglobin concentration (*r*=−0.59, *P*=0.02).

### 3.3. The relationship between proBDNF and immune function

At baseline, proBDNF was correlated with IL-8 (*r*=−0.58, *P*=0.05). At 12 months follow-up, proBDNF was correlated with IL-8 (*r*=0.69, *P*=0.01), IL-1α (*r*=0.78, *P*<0.01), IL-1β (*r*=0.78, *P*<0.01), VEGF (*r*=0.64, *P*=0.02), and EGF (*r*=0.86, *P*<0.001). proBDNF was correlated with CD16+56+ (%) (*r*=−0.78, *P*<0.01) and a trend was noted for CD3+CD4+ (cells/µL) (*r*=0.56, *P*=0.06). A statistical trend was noted for the correlation between proBDNF and red cell distribution width (*r*=−0.55, *P*=0.06).

### 3.4. The relationship between BDNF/proBDNF ratio and immune function

At baseline, BDNF/proBDNF ratio was correlated with VEGF (*r*=0.72, *P*<0.01) and EGF (*r*=0.59, *P*<0.051), and trends were noted with IL-1β (*r*=0.54, *P*=0.07), TNFα (*r*=0.55, *P*=0.07), and MCP-1 (*r*=0.52, *P*=0.08). BDNF/proBDNF ratio was correlated with CD3+ (%) lymphocytes (*r*=−0.72, *P*<0.01), CD3+CD4+ (%) (*r*=−0.72, *P*<0.01), CD19+ (%) (*r*=0.63, *P*=0.03), and CD19+ (cells/µL) (*r*=0.64, *P*=0.03), and a trend was noted for CD16+56+ (%) (*r*=0.52, *P*=0.08). BDNF/proBDNF ratio was correlated with red blood cells (*r*=0.68, *P*=0.02), hemoglobin (*r*=0.57, *P*=0.05), and hematocrit (*r*=0.57, *P*=0.05), and a trend was noted for mean platelet volume (*r*=−0.53, *P*=0.07).

At 12 months follow-up, BDNF/proBDNF ratio was correlated with VEGF (*r*=0.58, *P*=0.05) and a trend was noted for EGF (*r*=0.57, *P*=0.06). BDNF/proBDNF ratio was correlated with CD95+CD3+ (%) lymphocytes (*r*=−0.77, *P*=0.03), CD95+CD3+ (cells/µL) lymphocytes (*r*=−0.78, *P*=0.02), and CD95+CD90+ (cells/µL) (*r*=0.70, *P*=0.05), and a trend was noted for CD14+CD95+ (%) (*r*=−0.65, *P*=0.08). BDNF/proBDNF ratio was correlated with mean corpuscular hemoglobin concentration (*r*=−0.66, *P*=0.02).

### 3.5. The stratification of BDNF to assess inflammation and immune function

Based on our prior work, we dichotomized participants at a BDNF level above and below 5000 pg/mL. Those subjects below 5000 pg scored lower than those subjects at or above 5000 pg on several biomarkers, including VEGF (t[14]=2.8, *P*=0.02), EGF (t[14]=2.3, *P*=0.05), CD95+CD3+ (%) (t[10]=3.7, *P*=0.01), CD95+CD3+ (cells/µL) (t[10]=2.5, *P*=0.03), CD14+CD95+ (%) (t[10]=3.6, *P*=0.01), platelets (t[14]=2.5, *P*=0.03), and neutrophils (cells) (t[14]=2.3, *P*=0.04). Table 5 displays the descriptive data for significantly different values.

**Table 5:** Inflammatory markers and immune function by BDNF level.

Variable	BDNF <5000 pg/mL	BDNF ≥5000 pg/mL	Significance level
VEGF (pg/mL)	17.1±13.7	44.7±24.8	*P*=0.02

EGF (pg/mL)	2.9±5.8	21.4±21.9	*P*=0.05

CD95+CD3+ (%)	18.7±8.4	4.2±2.7	*P*=0.01

CD95+CD3+ (cells/µL)	338.4±202.8	94.8±74.7	*P*=0.03

CD14+CD95+ (%)	29.8±10.4	11.7±4.9	*P*=0.01

Platelets (#)	234.3±45.6	313.5±77.3	*P*=0.03

Neutrophils (cells)	3.3±1.2	4.9±1.5	*P*=0.04

Descriptive data are mean±standard deviation. EGF: Epidermal growth factor, VEGF: Vascular endothelial growth factor, BDNF: Brain-derived neurotrophic factor

## 4. Discussion

The current study shows a significant relationship between BDNF and several markers of immune functioning, particularly VEGF and EGF. These findings may be interesting and ultimately clinically useful, as it is known that these growth factors generally play an important role in T-cell proliferation, B-cell activation and class switching, local inflammation with endothelial activation, and vascular growth [30-34]. The data also showed a potential interrelationship between EGF and BDNF and proBDNF. EGF was significantly correlated with BDNF and the BDNF/proBDNF ratio at baseline and BDNF and proBDNF at 12 months. This suggests BDNF’s role in EGF-induced proliferation of neurogenesis and synaptic plasticity. The extant literature has shown potential mechanisms by which this may occur, including the PI3K/Akt pathway and the activation of neuronal protease m-calpain [35,36].

The correlations between BDNF, proBDNF, and VEGF were also compelling. Our results showed that at baseline VEGF correlated with levels of the BDNF/proBDNF ratio. At 12 months follow-up, significant relationships were noted among VEGF and proBDNF, BDNF, and the BDNF/proBDNF ratio. Experimental studies have found that both BDNF and VEGF play an important role in cell survival, neuroplasticity, hippocampal neurogenesis, and neural protection during infarction and inflammation from oxidative stress [37,38]. In addition, ongoing research has shown that neural stem cell transplantation in a transgenic mouse model of AD improved spatial learning and memory function through increased levels of neurotrophic factors and VEGF [39]. Our results support this important connection between these two immune modulators.

CD95+ cells may also play an important role in neuronal destructive and reparative mechanisms. These cells have been shown in mice to aggravate the degenerative processes in the acute phase, but induce regenerative processes in the repair phase of pneumococcal meningitis [40]. Furthermore, the role of CD95+ in apoptosis has been well-established and has been further extrapolated to include the tuning of neuroplasticity [41]. In AD, CD95+ cells have been implicated in calpain-induced cell damage and neurocognitive decline [42,43]. Our results show an additional relationship with BDNF, suggesting that this neurotrophic factor may exert effects through this mechanism.

BDNF may help mitigate neural destruction through the role of NK cells, such as CD16+CD56+. The role of CD16+CD56+ in chronic diseases, such as coronary artery disease, hemophagocytic lymphohistiocytosis, and arthritis, has been well-established [44-46]. Yet, CD16+CD56+ cells also play a key role in AD, as they increase apoptosis [47]. Our results showing a relationship between BDNF and this type of NK cell may extend an additional understanding of how BDNF exerts its effect.

Other recent findings have suggested an involvement between BDNF and altered platelet functioning in the pathogenesis of AD [48], which were supported by the results of the current study, as platelets were found to be significantly associated with BDNF. Our study also showed an inverse relationship between neutrophils and BDNF. In transgenic AD models, other studies have shown that neutrophil depletion or inhibition of neutrophil trafficking has reduced AD and improved memory in mice, already showing signs of cognitive dysfunction [49]. These relationships can help researchers further understand the role of inflammatory markers and immune function, as it relates to levels of BDNF in people living with AD.

### 4.1. Limitations

The current study is limited by small sample size. With a larger sample size, perhaps even more evidence could have been collected to evaluate the relationship between proBDNF and BDNF and inflammation and immune function. While we did not formally utilize a compliance measure in the study, caregivers were highly motivated to follow the protocol, given that these subjects were typically ineligible for other research studies, due to the severity of their impairment. Furthermore, all subjects were either attending the daily daycare program or were residents at the medical center, so the study staff was able to have regular contact with the caregiver and to reinforce the need to be compliant to the protocol. As with any study that attempts to elucidate physiological relationships during the course of an intervention, it can be challenging to interpret the results especially in light of other factors that were not accounted for, such as the effects of overall diet, physical activity level, caregiver support, and polypharmacy. Certainly, future studies should attempt to include nutrition factors, dietary habits, and physical activity/fitness levels as much as possible. In addition, confounding from likely different comorbid complications in this impaired sample cannot be entirely ruled out in a study such as this. The extent to which our conclusions are validated is impacted by both the efficacy of the APMC and whether we posited the right hypotheses. More research into this question is warranted along with studying a larger sample of participants.

## 5. Conclusions

Current insights into how the brain functions are still evolving. The relationship between neurological functioning according to BDNF and inflammation and immune functioning is also incomplete and needs further study. Our results demonstrate several interesting relationships between BDNF and proBDNF and markers of inflammation and immune function, i.e., VEGF, EGF, and CD95+ cells, which help to shed light on the links between the brain and the immune system. Thus, future studies down this line of research might help to underscore the important role of the immune system on neurological function.
